# *N*-(4-bromophenethyl) Caffeamide Protects Skin from UVB-Induced Inflammation Through MAPK/IL-6/NF-κB-Dependent Signaling in Human Skin Fibroblasts and Hairless Mouse Skin

**DOI:** 10.3390/molecules22101639

**Published:** 2017-09-29

**Authors:** Yueh-Hsiung Kuo, Po-Yuan Wu, Chien-Wen Chen, Ping Lin, Kuo-Ching Wen, Chien-Yih Lin, Hsiu-Mei Chiang

**Affiliations:** 1 Department of Chinese Pharmaceutical Sciences and Chinese Medicine Resources, China Medical University, Taichung 404, Taiwan; kuoyh@mail.cmu.edu.tw; 2 Department of Biotechnology, Asia University, Taichung 413, Taiwan; yihlin@asia.edu.tw; 3 Department of Dermatology, China Medical University Hospital, Taichung 404, Taiwan; wu.poyuan@gmail.com; 4 School of Medicine, China Medical University, Taichung 404, Taiwan; 5 Department of Cosmeceutics, China Medical University, Taichung 404, Taiwan; walnut0727@hotmail.com (C.-W.C.); a50535l@hotmail.com (P.L.); kcwen0412@gmail.com (K.-C.W.)

**Keywords:** *N*-(4-bromophenethyl) caffeamide, photoinflammation, propolis, IL-6, NF-κB

## Abstract

Long-term exposure to ultraviolet (UV) irradiation causes skin inflammation and aging. *N*-(4-bromophenethyl) caffeamide (K36H) possesses antioxidant and antimelanogenic properties. The present study investigated the effects of K36H on UVB-induced skin inflammation in human skin fibroblasts and hairless mice and evaluated the underlying mechanisms. The *in vitro* results indicated that K36H reduced UVB-induced mitogen-activated protein kinase (MAP kinase) expression. Furthermore, K36H treatment reduced cyclooxygenase-2 (COX-2) and inducible nitric oxide synthase (iNOS) protein expression in UVB-irradiated fibroblasts by regulating IκB and nuclear factor-kappa B (NF-κB) expression. In the animal study, topically applied K36H markedly reduced inflammation and skin thickness and prevented photodamage to the skin of hairless mice. In addition, K36H inhibited the levels of UV-upregulated inflammation-related proteins levels such as IL-1, iNOS, and NF-κB in the dermis of hairless mice. Our findings demonstrated the antioxidant and anti-inflammatory properties of K36H in human skin fibroblasts and hairless mice. Therefore, K36H can be developed as an antiphotodamage and antiphotoinflammation agent.

## 1. Introduction

Exposure to sunlight can cause oxidative stress and inflammation, resulting in melanogenesis and mutations in skin cells; furthermore, sun exposure can result in skin disorders and cancer [1]. Several studies have indicated that both ultraviolet (UV) A and UVB can cause skin photodamage by stimulating reactive oxygen species (ROS) generation, activating downstream signaling and inflammatory cytokine formation [2]. These cytokines subsequently activate mitogen-activated protein (MAP) kinase signaling and modulate downstream transcription factors, resulting in an inflammatory response [3]. Nuclear factor-κB (NF-κB) is a transcription factor that modulates the gene expression of various proinflammatory factors, chemokines, and growth factors. NF-κB and IκB exist as an inactive complex in the cytoplasm. Some proinflammatory factors and MAP kinases activate IκB kinase, triggering IκB ubiquitination. Subsequently, NF-κB is translocated to the nucleus, where it regulates inflammation-related gene transcription and protein expression, leading to inflammation and erythema of the skin [4,5]. Furthermore, UV irradiation leads to the generation of ROS that attack biomembrane lipids, increase prostaglandin E2 and nitric oxide secretion, and upregulate prostaglandins, interleukins (ILs), and other cytokines, causing inflammation of the skin [6,7,8]. In addition, MAP kinases directly activate NF-κB and promote the translocation of these transcription factors to the nucleus, causing skin inflammation and related skin disorders [7,9].

**Figure 1:**
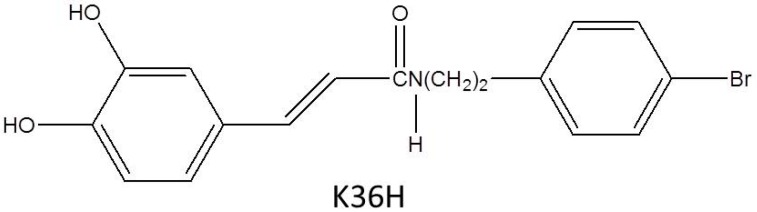
The structure of *N*-(4-bromophenethyl) caffeamide (K36H).

Materials with antioxidant and anti-inflammatory properties can be used to prevent premature skin aging [10,11,12]. *N*-(4-bromophenethyl) caffeamide (K36H, also named EK5, Figure 1) is a caffeic acid phenethyl ester (CAPE) derivative present in propolis. Previous studies have reported that CAPE possesses antioxidant, anti-inflammatory, antiviral, anticarcinogenic, and immunomodulatory properties [13,14]. Our previous studies revealed a caffeamide derivative with antiphotoaging properties [15,16]. K36H was discovered to inhibit melanogenesis and tyrosinase activity in B16F0 cells [17]. In addition, K36H downregulated NF-κB signaling in human monocytic cells and therefore exhibited anti-inflammatory properties [13].

UV light exposure induces skin damage. UVB irradiation is mainly responsible for severe damage such as sunburn, skin damage, and skin cancer [18]. UVA penetrates deeper into the skin than UVB. UVA is not directly absorbed by biological targets and can cause skin and tissue damage [19]. Although UVB radiation only constitutes approximately 5% of the total solar UV irradiation, it can cross the entire epidermis layer and penetrate the dermis compartment of human skin and has been reported to be a major cause of skin inflammation and photodamage [20]. Because no study has investigated whether K36H ameliorates skin inflammation and UVB-induced premature skin aging, we examined the antiphotodamage effects of K36H and the underlying mechanisms in human Hs68 cells by using an animal model.

## 2. Results

### 2.1. Effect of K36H on Cytotoxicity

**Figure 2:**
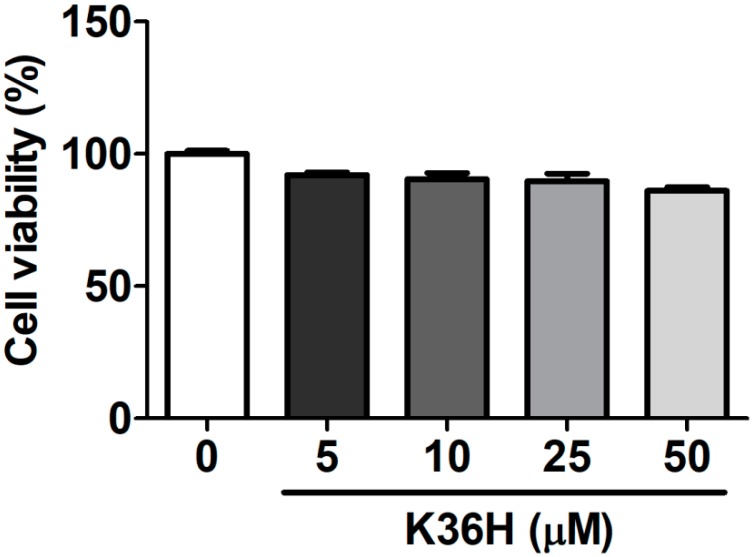
Human skin fibroblast viability (%) after treatment with K36H. K36H was not toxic to Hs68 cells.

Hs68 cells were treated with 5–50 μM K36H for 24 h, and cell viability was measured using MTT assay. The survival rate of Hs68 cells treated with 50 μM K36H was 86.1 ± 1.4% (Figure 2). Cell viability of ≥80% has been reported to be the criterion for cytotoxicity [21,22]. Therefore, the survival rate indicated that K36H (5–50 μM) was not toxic to Hs68 cells.

### 2.2. Antioxidant Properties of K36H

#### 2.2.1. K36H Scavenged DPPH Radicals

**Figure 3:**
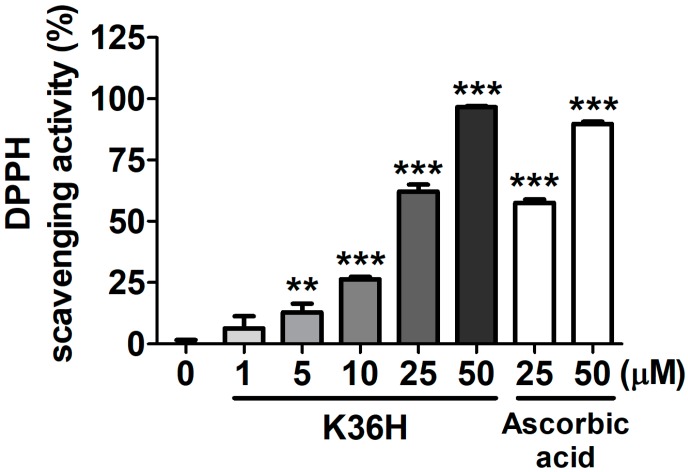
1,1-Diphenyl-2-picrylhydrazyl (DPPH) radical scavenging activity of K36H. Significant difference versus nontreatment group: **, *p* < 0.01; ***, *p* < 0.001.

The DPPH radical scavenging activity assay is generally employed as a model to evaluate the antioxidant properties of target drugs for free radical scavenging [23]. K36H exhibited a scavenging activity of 62.1 ± 2.9% and 96.6 ± 0.4% at 25 and 50 μM, respectively. The scavenging activity of K36H was superior to that of equal concentrations of ascorbic acid (Figure 3). The SC_50_ of K36H for DPPH scavenging activity was 20.0 ± 1.0 μM.

#### 2.2.2. K36H Alleviated UVB-Induced ROS Generation in Hs68 Cells

**Figure 4:**
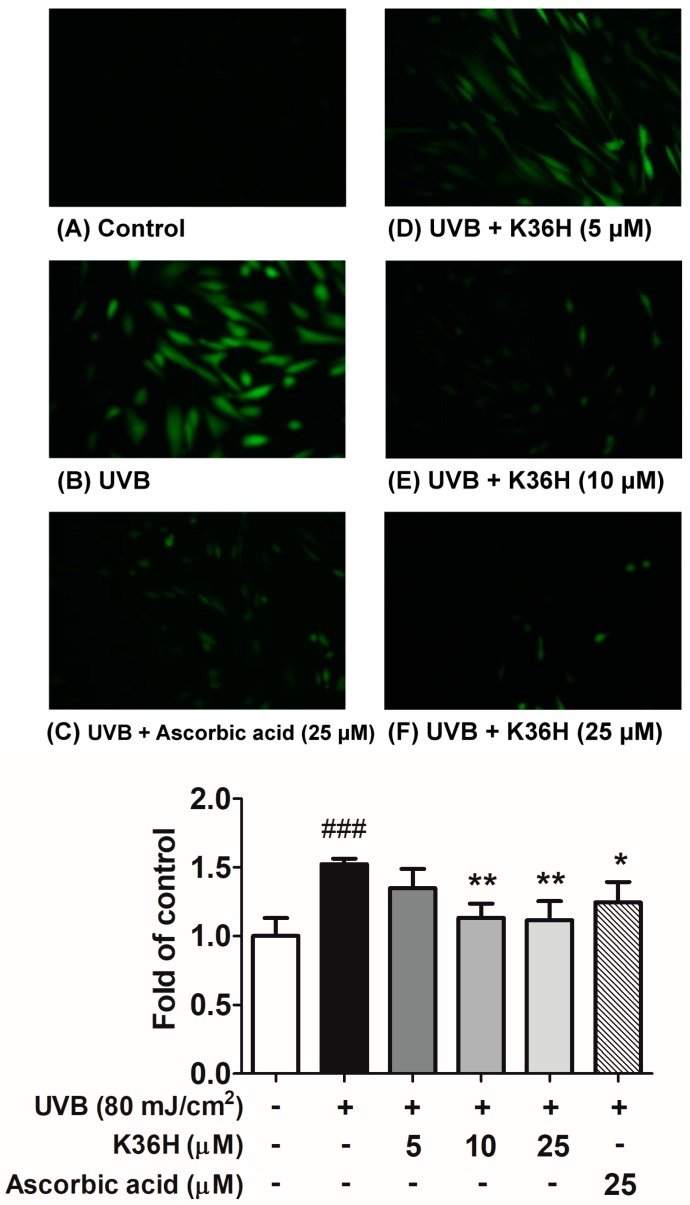
Intracellular reactive oxygen species (ROS) level measurement with 2′,7′-dichlorofluorescin diacetate (DCFDA) reagent after K36H treatment in ultraviolet (UV) B–irradiated human fibroblasts. Ascorbic acid was used as positive control. Significant difference versus nonirradiation group: ^###^, *p* < 0.001. Significant difference versus nontreatment group: *, *p* < 0.05; **, *p* < 0.01.

UV-induced ROS generation can trigger skin photoaging-related gene and protein expression and downstream signal transduction, causing oxidative stress in human skin cells. In this study, treatment with 80 mJ/cm^2^ UVB considerably increased ROS generation in Hs68 cells, whereas treatment with UVB and 5, 10, and 25 μM K36H resulted in 80%, 90%, and 100.0% less ROS formation, respectively (Figure 4). Ascorbic acid inhibited the intracellular ROS generation induced by UVB. Therefore, K36H alleviated UVB-induced damage by scavenging ROS in the Hs68 fibroblasts.

### 2.3. K36H Inhibited MAP Kinase Expression

**Figure 5:**
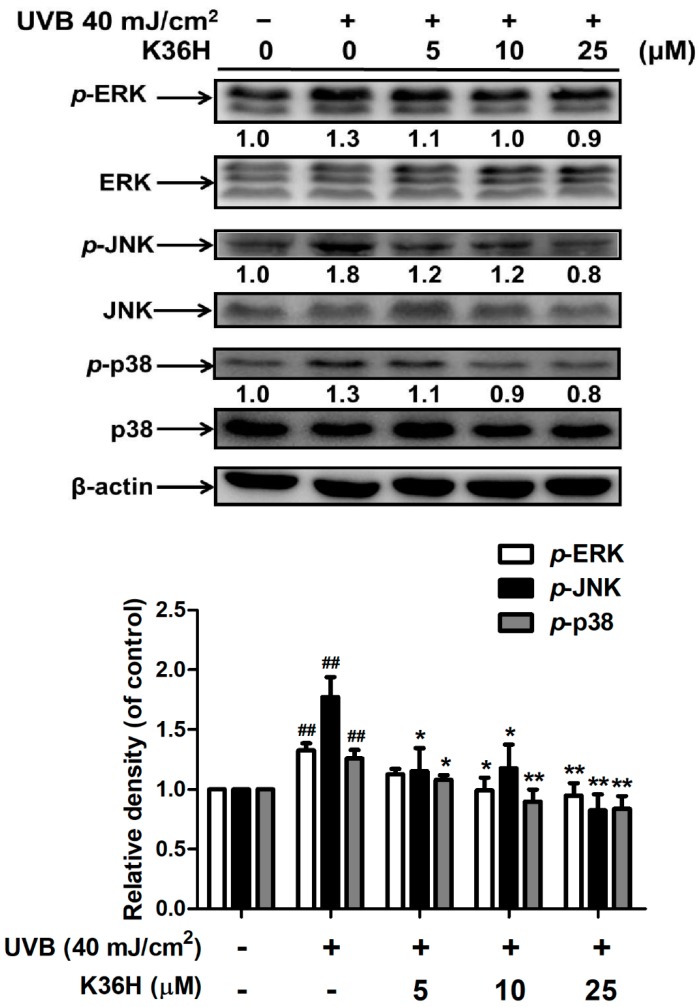
Effect of K36H on the UVB-induced phosphorylation of mitogen-activated protein (MAP) kinases in human fibroblasts. Significant difference versus nonirradiation group: ^##^, *p* < 0.01. Significant difference versus nontreatment group: *, *p* < 0.05; **, *p* < 0.01.

UVB irradiation induced the phosphorylation of MAP kinases, triggering downstream signal transduction and resulting in the regulation of the MMP expression level. After UVB irradiation, the phosphorylation of ERK increased 1.3-fold compared with the control group, and this effect was diminished significantly by K36H treatment at >10 μM concentrations for 24 h. The results for JNK and p-38 phosphorylation were similar to those for ERK. K36H treatment at >10 μM concentrations reduced UVB-induced JNK phosphorylation and p-38 expression (Figure 5).

### 2.4. K36H Alleviated UVB-Induced Inflammation in Human Skin Fibroblasts

#### 2.4.1. K36H Reduced UVB-Induced iNOS and COX-2 Overexpression

**Figure 6:**
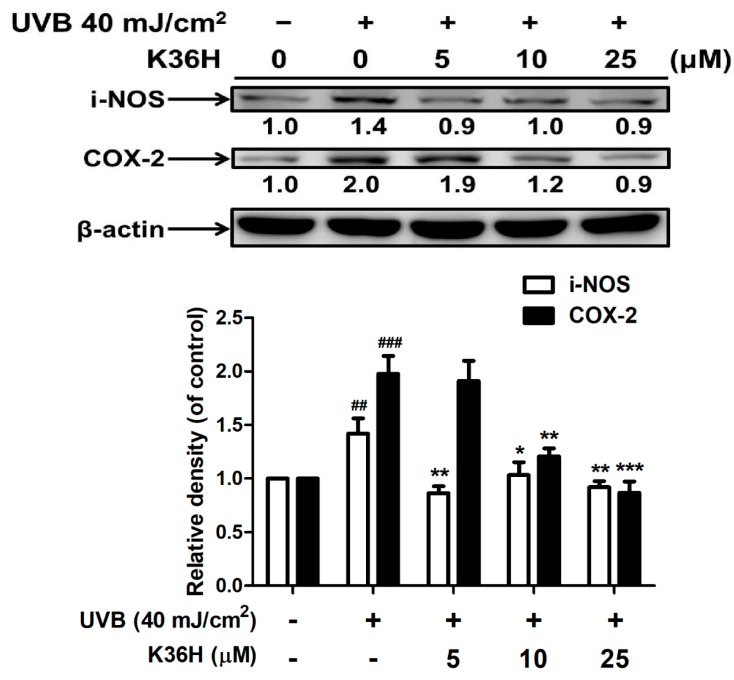
UVB-induced inducible nitric oxide synthase (iNOS) and COX-2 expression levels in human fibroblasts. Significant difference versus nonirradiation group: ^##^, *p* < 0.01; ^###^, *p* < 0.001. Significant difference versus nontreatment group: *, *p* < 0.05; **, *p* < 0.01; ***, *p* < 0.001.

Figure 6 illustrates the effects of K36H treatment on iNOS expression in Hs68 cells. K36H dose-dependently inhibited UVB-induced iNOS overexpression. UVB irradiation increased iNOS expression (by 1.4-fold compared with the control group); however, 24 h treatment with 5 μM K36H significantly reduced iNOS expression by 0.9-fold compared with the control group.

UVB irradiation increased COX-2 expression in Hs68 cells by 2.0-fold compared with the control group (Figure 5). Furthermore, K36H treatment (5–25 μM) dose-dependently reduced UVB-induced COX-2 expression levels; this effect was significant at >10 μM concentrations (Figure 6).

#### 2.4.2. K36H Modulated IκB/NF-κB Transduction

**Figure 7:**
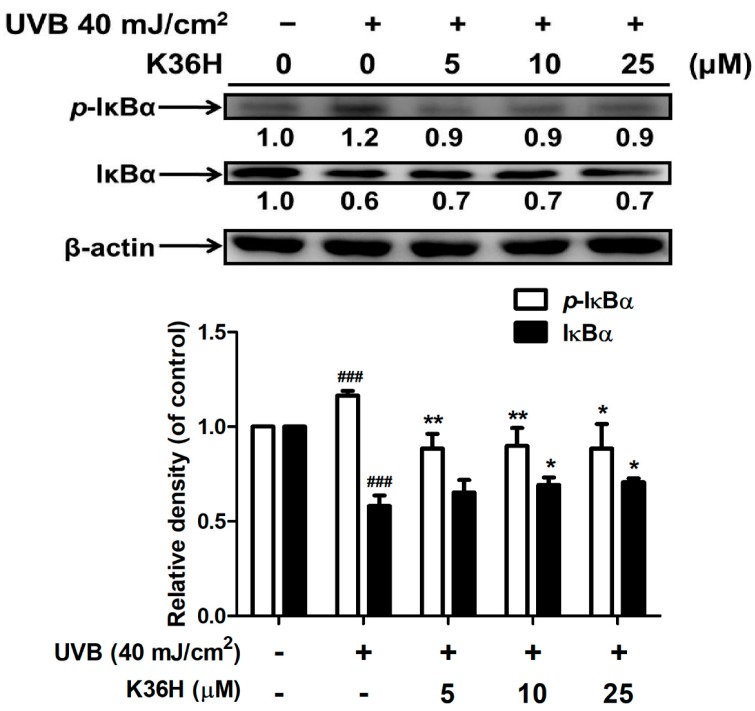
Effect of K36H on UVB-induced p-IκBα and IκBα expression in human fibroblasts. Significant difference versus nonirradiation group: ^###^, *p* < 0.001. Significant difference versus nontreatment group: *, *p* < 0.05; **, *p* < 0.01.

The results of Western blotting indicated that UVB irradiation suppressed IκBα expression but increased p-IκBα expression. Treatment with 5 μM K36H for 24 h increased p-IκBα expression by 1.2-fold but significantly reduced p-IκBα expression by 0.9-fold compared with the control group (Figure 7). In addition, treatment with 10 μM K36H significantly enhanced IκBα expression (Figure 7). IκBα is degraded because of ubiquitination; thus, NF-κB is translocated from the cytoplasm to the nucleus, causing an inflammatory response.

**Figure 8:**
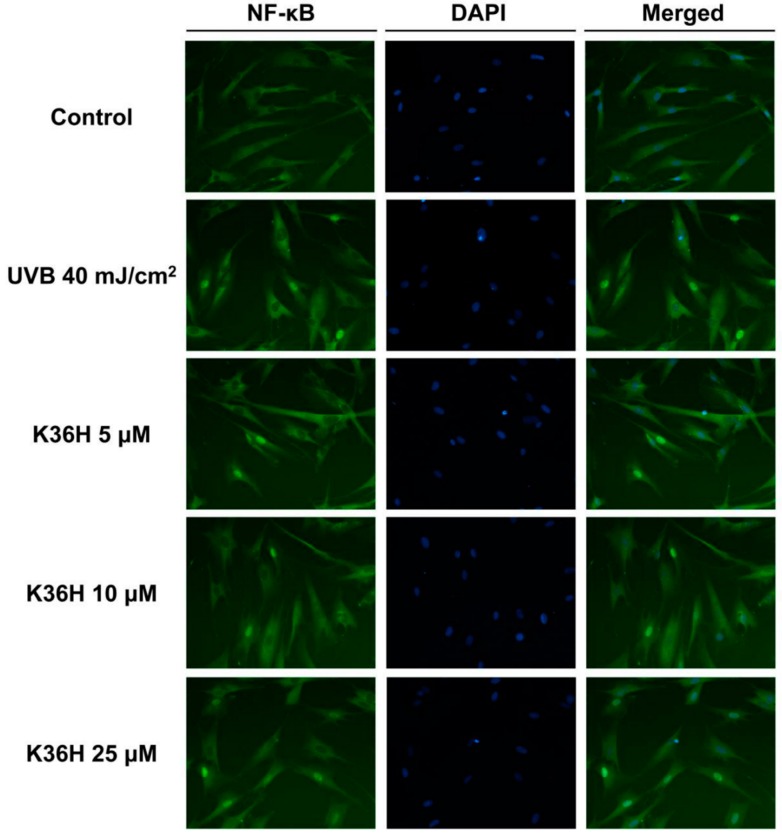
Effect of K36H on UVB-induced activation of NF-κB P65 in human fibroblasts. K36H inhibited the UVB-induced translocation of NF-κB into the nucleus.

In this study, immunohistochemical staining of NF-κB in the fibroblasts was applied to assay the activation of NF-κB. As demonstrated in Figure 8, UVB irradiation triggered NF-κB translocation to the nucleus, whereas K36H treatment inhibited this effect. K36H inhibited the ubiquitination of IκBα, thus preventing the activation of NF-κB and skin photodamage.

### 2.5. Effects of K36H on UVB-Induced Skin Inflammation in Hairless Mice

UVB exposure resulted in skin inflammation and elevated a* values of the skin. In the present study, UVB increased the a* values, whereas K36H reduced the a* values without significant differences (data not shown).

**Figure 9:**
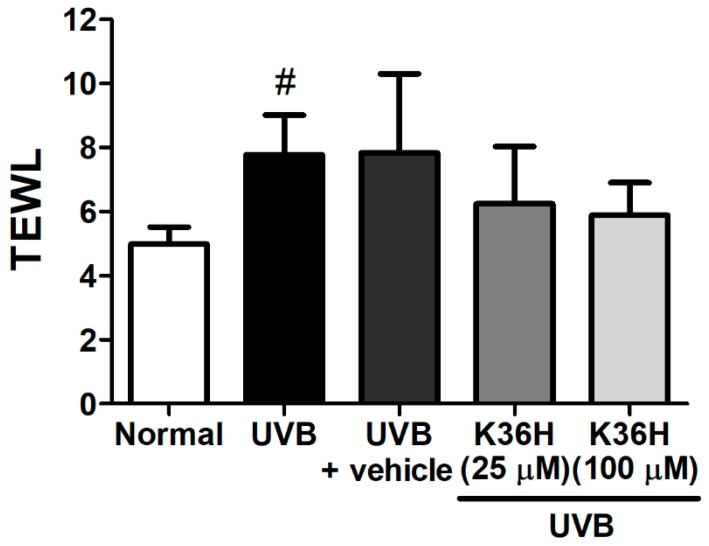
Effect of K36H on transepidermal water loss (TEWL) in chronic UVB-irradiated hairless mice at 12 weeks.

Transepidermal water loss (TEWL) is an indicator of skin barrier function. UVB exposure disturbed the skin barrier function, thereby increasing TEWL in mice skin. UV exposure caused significant changes in the TEWL of hairless mice dorsal skin. However, TEWL did not exhibit significant differences after K36H application on the dorsal skin of hairless mice for 12 weeks (Figure 9). These results suggested that K36H did not exhibit skin toxicity or disturb the skin barrier function.

### 2.6. K36H Reduced UVB-Induced Epidermal Hyperplasia in Hairless Mouse Skin

**Figure 10:**
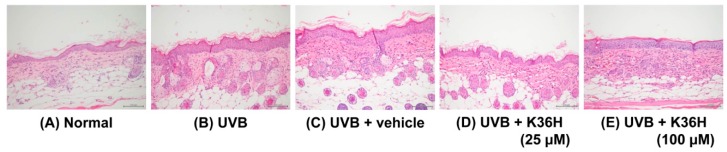
Light micrographs of histological sections stained with hematoxylin and eosin (H&E) in hairless mice.

**Figure 11:**
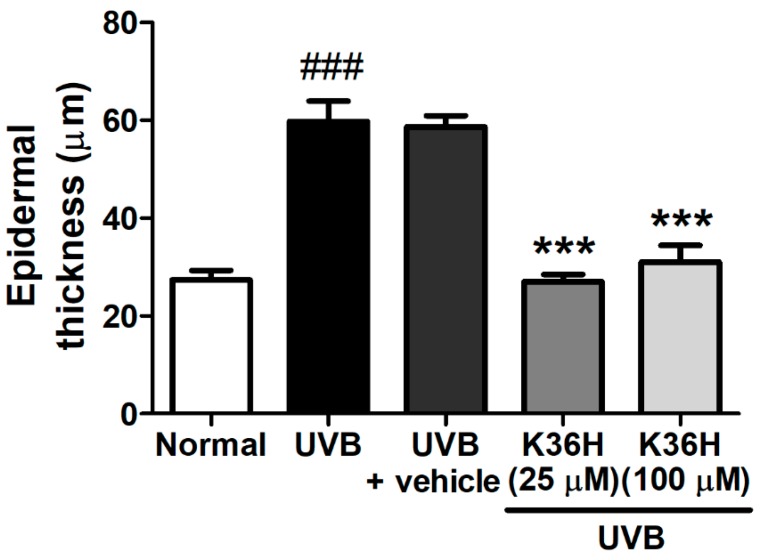
Effect of K36H on the epidermal thickness in UVB-exposed hairless mice. Significant difference versus normal group: ^###^ *p* < 0.001. Significant difference versus UVB group: *** *p* < 0.001.

The epidermal thickness of UVB-irradiated mice increased significantly after 12 weeks (Figure 10 and Figure 11). However, K36H application on the dorsal skin reduced this UVB-induced epidermal hyperplasia (Figure 10 and Figure 11).

### 2.7. Effects of K36H on Inflammation-Related Proteins in UVB-Exposed Mice Skin

**Figure 12:**
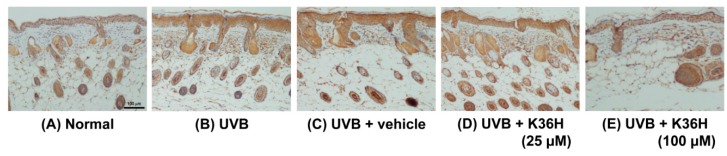
Immunohistochemical staining of IL-6 expression on mice skin slices. K36H inhibited UVB-induced IL-6 overexpression in mice skin.

**Figure 13:**
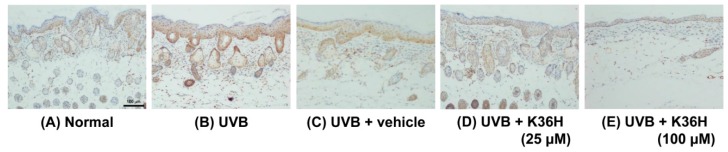
Immunohistochemical staining of iNOS expression on mice skin slices. K36H inhibited UVB-induced iNOS overexpression in mice skin.

**Figure 14:**
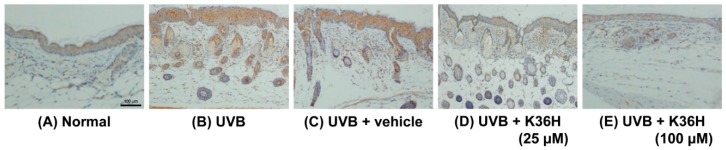
Immunohistochemical staining of NF-κB expression on mice skin slices. K36H inhibited UVB-induced NF-κB overexpression in mice skin.

Figure 12, Figure 13 and Figure 14 indicated that the expressions of IL-6, iNOS, and NF-κB were increased after 12-weeks UVB exposure; K36H treatment decreased the expression of these proteins. The results indicated that topically applied K36H inhibited UVB irradiation–induced inflammatory protein expression. Therefore, K36H inhibited UVB-induced inflammation and collagen degradation, exhibiting antiphotodamage properties.

## 3. Discussion

UV-induced oxidative stress and inflammation cause skin damage and aging. ROS production is initiated through a complex cascade of signal transductions and reactions in the skin. UV exposure increases ROS generation in skin cells, inducing skin damage and lipid peroxidation. Therefore, photodamage prevention strategies involve free radical scavenging and quenching. Furthermore, anti-inflammation agents such as COX and cytokine production inhibitors may prevent skin photodamage [24,25,26]. Accordingly, antioxidant and anti-inflammatory agents can be applied to alleviate photoaging [27,28]. The results of this study indicated that K36H quenches DPPH radicals and reduces UVB-induced ROS generation in Hs68 cells. However, it did not exhibit significant scavenging activity of hydrogen peroxide and ferrous chelating activity. A previous study demonstrated the antioxidant properties of CAPE, and its catechol group may contribute to its antioxidant properties [29]. The catechol groups of caffeamide derivatives are electron donors that terminate free radical activity. Exogenous antioxidant application may be an effective strategy to ameliorate ROS-induced skin damage, which is caused by excessive UV exposure [30,31].

UV irradiation leads to ROS generation, which promotes MAP kinase activation and recruits AP-1 to the nucleus and subsequently activates NF-κB and proinflammatory-related genes and proteins [32,33]. The generation of cytokines, such as IL-1 and IL-6, mediates UV-induced skin inflammation. UVB exposure activates NF-κB, which plays a key role in inflammation that is mediated through the activation of multifunctional cytokines such as IL-6 by binding to the promoter region [34,35]. UVB exposure induces oxidative stress that stimulates MAP kinases and NF-κB, ultimately increasing skin inflammation [36]. Activated NF-κB can induce MMP expression in dermal fibroblasts, causing skin damage and photoaging [34]. A study reported that kirenol reduced the UVB-induced phosphorylation of ERK, JNK, and p38 and inhibited the expression of inflammatory mediators (NF-κB, IL-6, and IL-8) to prevent UVB-induced inflammation and photodamage [36].

UV exposure causes inflammatory changes in the skin, such as erythema and hyperpigmentation, and the production of inflammatory cytokines and proteins that result in skin appearance and vascular responses [37]. *In vivo* UVB exposure of murine skin caused IL-1 production in epidermal cells, which was detected in UV-exposed human skin [38]. The secretion of these proinflammatory cytokines plays a pivotal role in the recruitment and activation of inflammatory cells, resulting in UVB-induced skin damage [29].

UV irradiation can cause NF-κB translocation, thereby inducing MMP production for collagen degradation in the dermis. The antioxidant activity of K36H may contribute to its inhibitory effect against NF-κB activation. K36H may prevent IκB dissociation from the complex with NF-κB. Furthermore, NF-κB modulates the protein expression and gene transcription of COX-2 and iNOS, leading to skin inflammation [30,32]. The present study demonstrated that K36H reduced UVB-induced COX-2 and iNOS protein expression in hairless mice. In addition, K36H inhibited the translocation of NF-κB to the nucleus in skin fibroblasts. K36H also inhibited the UVB-induced overexpression of IL-6, iNOS, and NF-κB in hairless mice, protecting them from UV-induced skin damage.

## 4. Materials and Methods

### 4.1. Materials

Thiazolyl blue tetrazolium bromide (MTT) and IGEPAL CA-630 were purchased from the USB Corporation (Cleveland, OH, USA). The medium, serum, and reagents for cell culture were obtained from Invitrogen Co. (Carlsbad, CA, USA). Other reagents used in this study were of reagent grade and were purchased from Sigma-Aldrich (St. Louis, MO, USA). K36H was supplied by one of the authors, Professor Kuo, and was synthesized as described previously [13].

### 4.2. Detecting 1,1-Diphenyl-2-picrylhydrazyl Radical Scavenging Activity

The 1,1-diphenyl-2-picrylhydrazyl (DPPH) radical scavenging activity of K36H was measured as described previously [15,39]. The positive control was ascorbic acid. Reaction mixtures containing DPPH prepared in methanol and 2–100 μM K36H were incubated at room temperature. Thereafter, the absorbance at 492 nm was detected on a microplate reader (Tecan, Untersbergstraße, Austria).

### 4.3. Fibroblast Culture and UV Exposure

Human foreskin fibroblasts (Hs68) were incubated in Dulbecco’s modified Eagle’s medium containing 10% fetal bovine serum and 100 U/mL penicillin and streptomycin. The cells were grown in an incubator at 37 °C with 5% CO_2_. The cells were exposed to UVB in a UV crosslinker (302 nm UV, 5 × 8 watt UV dual bipin discharge type, CL-1000M, UVP, Upland, CA, USA) that delivered a UVB energy of wavelength 302 nm. The UVB dose was 40 mJ/cm^2^, and the exposure was approximately 15 s.

### 4.4. Intracellular ROS Assay

The intracellular ROS in the Hs68 cells were detected using the 2′,7′-dichlorofluorescin diacetate (DCFDA) reagent, as previously described [40,41]. Ascorbic acid (25 μM) was used as positive control. The fibroblasts were exposed to UVB irradiation and incubated with 5–25 μM K36H for 1 h. Thereafter, DCFDA was added, and the mixture was incubated for 30 min. The fluorescence of the mixture (emission and excitation wavelengths: 488 and 520 nm, respectively) was assayed on an enzyme-linked immunosorbent assay reader (Thermo Electron Corporation, Vantaa, Finland). Images were captured using a fluorescence microscope (Leica DMIL, Heidelberg, Germany).

### 4.5. Western Blotting

The fibroblasts were exposed to UVB irradiation and incubated with 5–25 μM K36H for 4 or 24 h. After UVB irradiation and K36H treatment, whole cell protein lysate was obtained for immunoblot analysis through Western blotting, as previously described [16]. The cell lysate was separated using sodium dodecyl sulfate polyacrylamide gel electrophoresis. Blots were blocked for 2 h with non-fat milk in TBS buffer. The membrane was incubated for 16 h with specific antibodies. The membranes were washed with TBST for 40 min. The blot was then incubated with the corresponding conjugated anti-immunoglobulin G-horseradish peroxidase (Santa Cruz Biotechnology Inc., Santa Cruz, CA, USA). Immunoreactive proteins were detected using specific antibodies with the enhanced chemiluminescent Western blotting detection system (Amersham, Buckinghamshire, UK), and the densitometric program was used to quantify band densities.

### 4.6. Immunofluorescence Staining

The cells were cultivated on cover slips and incubated with 5–25 μM K36H for 24 h after UVB exposure, as previously described [15]. The cells were fixed with 4% paraformaldehyde for 30 min. The cells were incubated with primary and secondary antibodies (Alexa Fluor 488 anti-rabbit IgG, Invitrogen, CA, USA). The cells were counterstained with ProLong® Gold antifade reagent, and images were obtained using a Confocal Spectral Microscope (Leica SP2, Frankfurt am Main, Germany).

### 4.7. Animal Study

The animal study was approved by the Institutional Animal Use and Care Committee of China Medical University, and the protocol number was 100-124-N. All methods were performed in accordance with the relevant guidelines and regulations. Six-week-old female BALB/c hairless mice were purchased from the National Laboratory Animal Center, Taipei, Taiwan. All mice were fed standard chow and kept in a temperature-, humidity-, and light/dark cycle–controlled room.

### 4.8. Effect of K36H on UVB-Induced Skin Inflammation

Six-week-old female BALB/c hairless mice were randomly divided into the following five groups (six per group): control group, UVB-irradiated group, UVB-irradiated and vehicle-treated group, UVB-irradiated and 25-μM-K36H-treated group, and UVB-irradiated and 100-μM-K36H-treated group. Mice were exposed to UVB irradiation (302 nm UV, 5 × 8 watt UV dual bipin discharge type, CL-1000M, UVP, Upland, CA, USA) three times per week and were treated with 50 μL of vehicle (glycerol) or K36H (25 or 100 μM) every day for 12 weeks, as previously described [42]. The UVB dose was 36 mJ/cm^2^ in the first week, 54 mJ/cm^2^ in the 2–4 weeks, 72 mJ/cm^2^ 5–7 weeks, and 108 mJ/cm^2^ 8–10 weeks [42].

Erythema and transepidermal water loss (TEWL) were measured using an MPA 580 system (Courage + Khazaka electronic GmbH, Cologne, Germany) [28].

### 4.9. Immunohistochemical Staining of Skin Slices

After 12 weeks of treatment, mice were sacrificed, and the dorsal skin samples were excised and fixed in 10% formaldehyde. The skin slices were stained with hematoxylin and eosin or Masson’s trichrome. The slices’ histopathology was examined using a microscope, and ImageJ software (Wayne Rasband, National Institutes of Health, Bethesda, MD, USA) was used to determine skin thickness. The samples were stained with specific monoclonal anti-mouse antibodies and examined under a microscope [28].

### 4.10. Data Analysis

Numerical data are expressed as mean ± standard deviation. All measurements in this study are expressed as the averages from at least three independent experiments. The group comparison results were analyzed using the Student’s *t* test or analysis of variance. All differences were statistically significant if *p* < 0.05.

## 5. Conclusions

The present study demonstrated that K36H inhibits UVB-induced skin inflammation by scavenging free radicals, inhibiting intracellular ROS generation, and suppressing overexpression of phosphorylated MAP kinases. In addition, K36H reduced iNOS and COX-2 expression and modulated the IκB/NF-κB pathway, attenuating photodamage to the skin. K36H ameliorated UV irradiation–induced wrinkle formation in the skin of hairless mice by inhibiting photoinflammation. To further understand the protective effects of K36H against UV-induced damage, additional experiments on the K36H-mediated prevention of UVA-induced skin damage are required in the future.
